# A-Site Cation Chemistry in Halide Perovskites

**DOI:** 10.1021/acs.chemmater.4c02043

**Published:** 2024-10-23

**Authors:** Matthew P. Hautzinger, Willa Mihalyi-Koch, Song Jin

**Affiliations:** † National Renewable Energy Laboratory, Golden, Colorado 80401, United States; ‡ Department of Chemistry, University of Wisconsin-Madison, Madison, Wisconsin 53706, United States

## Abstract

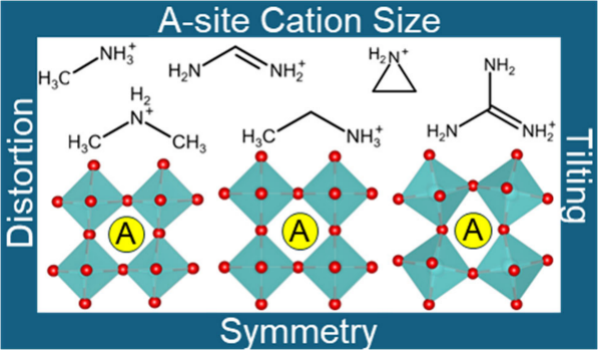

Metal halide perovskites are an important class of semiconductors now being implemented as photovoltaic absorbers and explored for light emission, among other device applications. The semiconducting properties of halide perovskites are deeply intertwined with their composition and structure. Specifically the symmetry, tilting, and distortions of the metal halide octahedra impact the band structure and other optoelectronic properties. In this review, we examine the various compositions of monovalent A-site cations in three-dimensional (3D) halide perovskites AMX_3_ (M = divalent metal; X = halide). We focus on how the A-site cation templates the inorganic metal-halide perovskite framework, resulting in changes in the crystal structure symmetry, as well as M–X bonding parameters, summarized in a comprehensive table of AMX_3_ structures. The A-site cation motion, effects of alloying, and 2D Ruddlesden–Popper perovskite structures with unique A-site cations are further overviewed. Correlations are shown between these A-site cation dominated structural parameters and the resulting optoelectronic properties such as band gap. This review should serve as a reference for the A-site cation structural chemistry of metal halide perovskites and inspire continued research into less explored metal halide perovskite compositions and structures.

## Introduction

Metal halide perovskites have numerous demonstrated semiconductor applications including high-efficiency photovoltaics (PVs),^1,2^ lasing and light emitting diodes,^3,4^ X-ray detectors,^5^ field effect transistors,^6^ and spintronic devices.^7,8^ Investigation into growing metal halide perovskites in thin films, nanocrystals (NCs), and other nanostructures and has enabled these applications.^9−11^ Underpinning such processing and applications are solid-state chemistry studies of the crystal structures and physical properties of halide perovskites.

**Figure 1:**
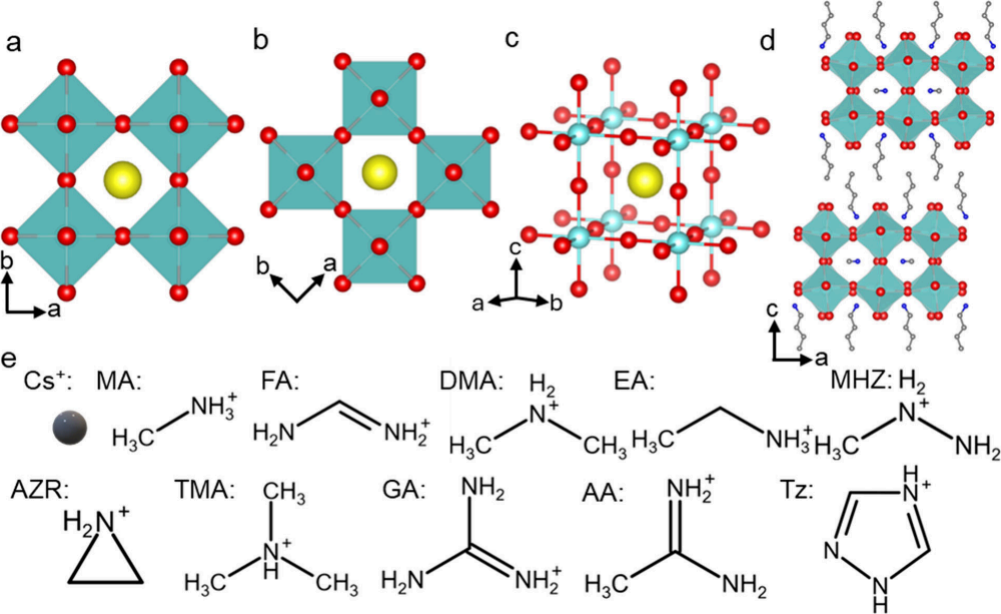
Common halide perovskite crystal structures and A-site cations. (a–c) Different representations of the 3D AMX_3_ structure with corner sharing metal halide octahedra. (d) Crystal structures of 2D Ruddlesden–Popper (RP) perovskite (*n* = 2). (e) A-site cations that have been confirmed via crystallography to occupy the A-site cavity of AMX_3_ or 2D (A′)_2_(A)_*n*−1_*M*_*n*_X_3*n*+1_ halide perovskite structures. MA = methylammonium, FA = formamidinium, DMA = dimethylammonium, EA = ethylammonium, MHz = methylhydrazinium, AZR = aziridinium, TMA = trimethylammonium, Ga = guanidinium, AA = acetamidinium, and Tz = 1,2,4-triazolium.

The prototypical three-dimensional (3D) halide perovskite structure AMX_3_ [A = monovalent cation; M = Pb^2+^, Sn^2+^, Ge^2+^; and X = I^–^, Br^–^, Cl^–^] is a corner sharing network of MX_6_^4–^ octahedra with an A-site cation occupying the 12-coordinate cavity formed by eight octahedra (Figure 1a–c), sometimes referred to as the “perovskite cage”.^12^ AMX_3_ halide perovskites are highly tunable with a variety of combinations of metal and halide ions, and their solid solutions are accessible. By incorporating bulky ammonium cations, the 3D MX_6_^4–^ octahedral network can be broken up into 2D layered perovskite variants with these bulky ammonium spacer cations between perovskite layers forming natural quantum wells (QWs), such as Ruddlesden–Popper (RP)^13,14^ (Figure 1d) or Dion–Jacobson (DJ)^15^ phases, with the formula (A′)_*m*_(A)_*n*−1_M_*n*_X_3*n*+1_ (A′ = alky- or aryl-ammonium spacer cation for *m* = 2 RP, or diammonium cation for *m* = 1 DJ perovskite). The number of inorganic layers (*n*), i.e., the thickness of the QWs, is controlled by the ratio of A-site and A′ cations.

The composition of the halide perovskite dictates the crystal symmetry, distortion of the perovskite framework, and the ability to form a stable perovskite. This in turn determines the optoelectronic properties. While several recent reviews have summarized the developments in the general structural chemistry and spacer cation tuning of 2D halide perovskites,^16−19^ the materials chemistry of A-site cations in 3D AMX_3_ perovskites has not been systematically surveyed from a solid-state chemistry point of view. In this Review, we will examine the role of the A-site cation in templating the inorganic framework through a comprehensive summary of the AMX_3_ crystal structures including the crystal symmetry, tilting of octahedra, and structural distortions. Select works on A-site cation motion and alloying of A-site cations will be discussed in the context of structural chemistry. We will also discuss the effect of A-site cations on higher *n*-value RP perovskites, specifically focusing on unique A-site cations that are not possible to incorporate in the 3D AMX_3_ phase. The relationship between the band gap energy and A-site cation is discussed as a proxy for how the A-site cation can impact the AMX_3_ structure.

### Description of Structural Tolerance Factor, Octahedral Tilting, and Distortions in Halide Perovskites

**Figure 2:**
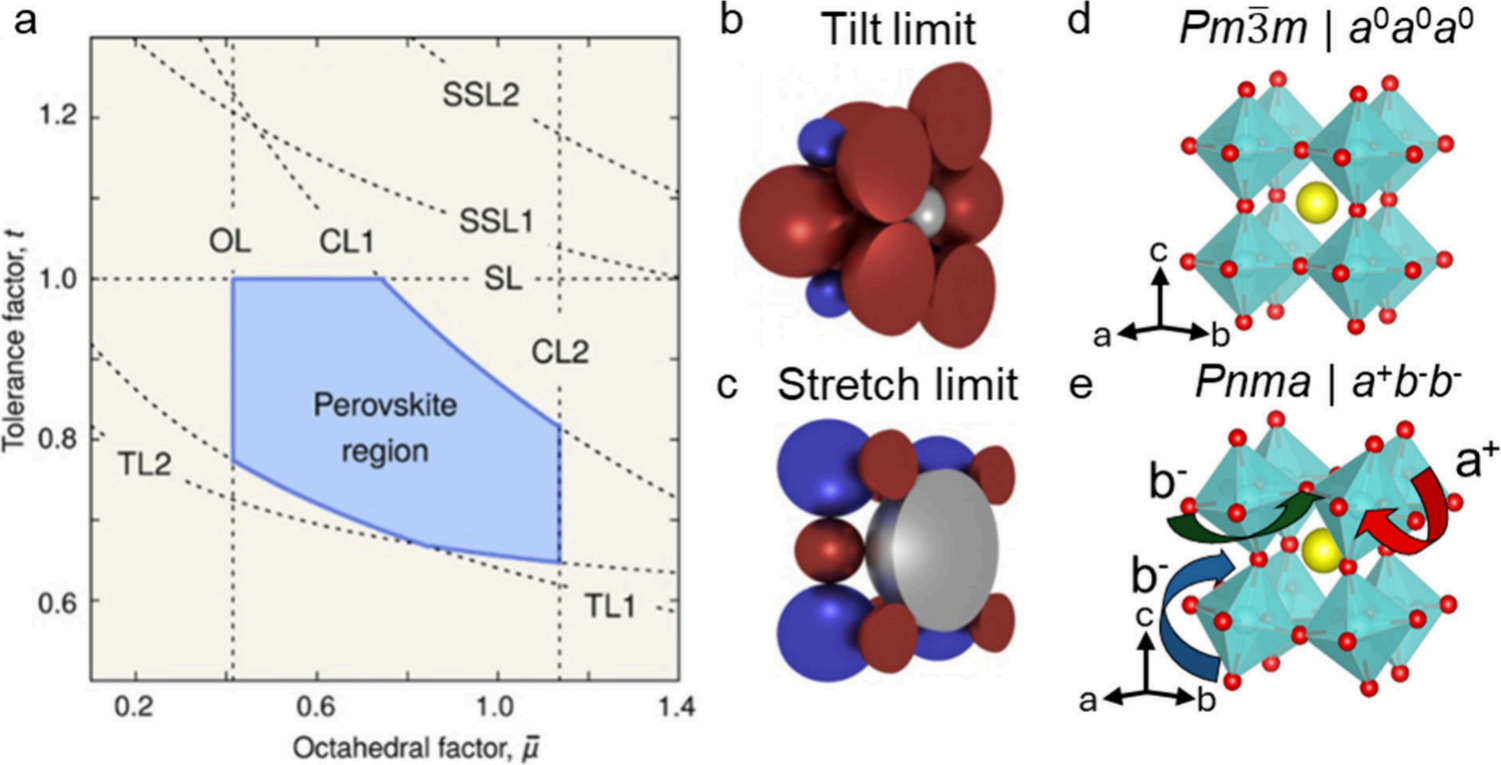
Description of the phase stability, octahedral tilting, and crystal symmetry in AMX_3_ halide perovskite. (a) Plot of tolerance factor and octahedral factor showing the blue shaded stability region of 3D perovskites as well as lines deliminating the tilt limit (TL), octahedral limit (OL), chemical limit (CL), stretch limit (SL), and secondary stretch limit (SSL). Schematic of the perovskite structures at the (b) tilt limit and the (c) stretch limit (silver = A-site cations, blue = B cations, and red = X anions). Representative structure diagram (d) of an *a*^0^*a*^0^*a*^0^ perovskite structure lacking octahedral tilting and an *a*^+^*b*^–^*b*^–^ structure (e) with tilting along the *a* axis (*a*^+^) in a different direction and lesser extent than the b and *c* axis tilting (*b*^–^). Panels a, b, and c reproduced from *Proc. Natl. Acad. Sci.***2018**, *115* (21), 5397–5402.^22^

Empirical models based on bonding and ionic radii have been developed to predict a perovskites’ ability to form.^20−24^ Relative to the traditional highly ionic oxide perovskites, the M–X bonds in halide perovskites are more covalent in nature.^23,24^ The empirical Goldschmidt tolerance factor (α) defined in eq 1 was originally used for highly ionic oxide perovskites and can be applied to the more covalent halide perovskites as a rule of thumb for whether a perovskite will form with a specific compsition.
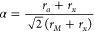
1*r*_*i*_ is the radii of the various ions in AMX_3_ (i = A, M, X). To determine *r*_*a*_ for the nonspherical organic cations, an effective ionic radius is calculated as the distance between the center of mass of the molecule to the furthest atom, added to the radius of that atom.^21^ In addition, the octahedral factor (μ) defined in eq 2 is used to predict the preferential formation of individual MX_6_ octahedra versus other geometries. The octahedral limit describes when the B metal is too small or large relative to the anions, resulting in formation of a different geometry such as a tetrahedron.
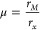
2Goldschmidt’s no-rattling principle dictates that α and μ must be within empirically defined limits for a stable perovskite to form, as shown in the plot of tolerance factor vs octahedral factor in Figure 2a.^22^

The size of the A-site cation plays a large role in dictating octahedral tilting.^22^ Under the tilt limit, the A-site cation is small, leaving a void for the octahedra to tilt at moderate α and promoting a nonperovskite structure when α < 0.8 (Figure 2b). In contrast, under the stretch limit where α > 1, the A-site cation is too large, stretching the M–X distances beyond what forms stable perovskites (Figure 2c). The structural symmetry is dictated by the type and degree of tilting of the octahedra. As described by the Glazer notation (e.g., *a*^0^*b*^+^*c*^–^), MX_6_ octahedra can tilt in the same direction (in-phase) or opposite direction (antiphase) or have no tilt, which is indicated along different crystallographic axis as superscripts + , −, and 0, respectively.^25^ Furthermore, the octahedra can have the same or unique amplitude of tilt, indicated by *a a a* for the same amplitude or *a b c* for each amplitude of tilt independent along unique crystallographic directions (ref (25) for complete description). For example, *a*^0^*a*^0^*a*^0^ has no tilting (Figure 2d) and adopts the *Pm*3̅*m* space group. In contrast, Figure 2e shows an *a*^+^*b*^–^*b*^–^ structure which has tilting about each axis, with the *b* and *c* axis the same degree of tilt, and fits into the lower symmetry *Pnma* space group.^26^

Another consideration is the individual MX_6_ octahedra, which can be distorted with deviations from the ideal M–X lengths and X–M–X bond angles. The bond length distortion index (*D*) describes the elongation of the M–X bond lengths (*d*_*i*_) relative to ideal M–X bond lengths (*d*_0_) in eq 3:^27^
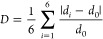
3The X–M–X bond angle (θ_*i*_) distortion can be described with the bond angle variance (σ^2^):^28^
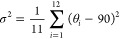
4The combination of *D* and σ^2^ captures the various distortions in an octahedron, which in turn alter the symmetry of the overall structure.

### Effect of A-Site Cations on the 3D Perovskite Structures

**Figure 3:**
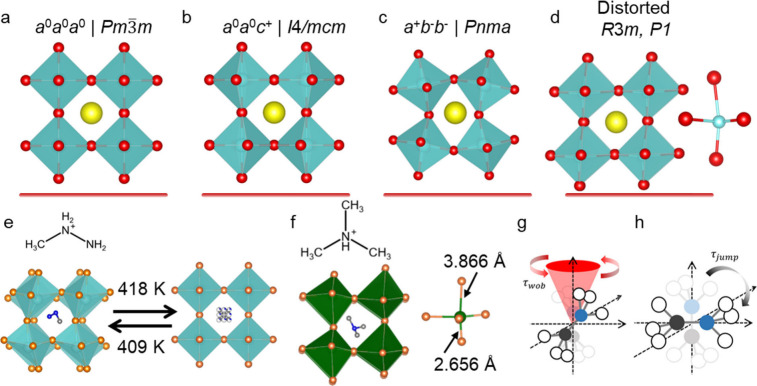
Range of halide perovskite structures from ideal to increasingly distorted, those with exotic A-site cations, and illustration of cation reorientations. (a) Ideal *Pm*3̅*m* perovskite structure with no tilting or distortion of the octahedra. (b) Octahedra slightly tilted along one axis due to the smaller ratio of A-cation size to MX_6_ (lower α). (c) Tilted perovskite where each axis is tilted. (d) Perovskite structure with distorted octahedra and an individual octahedron highlighted on the right. (e) The methylhydrazinium (MHz) cation found in the RT noncentrosymmetric *P*2_1_ and high temperature *Pm*3̅*m* structures of (MHz)PbBr_3_.^29^ (f) Trimethylammonium (TMA) cation and the (TMA)SnBr_3_ structure.^30,31^ The distortions and off-centering of Sn^2+^ can be clearly seen in the individual octahedron highlighted on the right. Diagrams illustrating the (g) wobbling rotations of MA cations and the (h) jump reorientation.^32^ Panels g and h are reproduced or adapted with permission from ref (32). Copyright 2018 American Chemical Society.

Halide perovskites form four common crystal structure types at room temperature (RT) depicted in Figure 3a–d depending on the interplay of ionic radius sizes among the A-site cation, metal, and halide. AMX_3_ compounds with crystal structures determined and their tolerance factors, symmetries, bonding parameters, and band gap of each compound are summarized in Table 1.

**Table 1:** Tolerance Factor (α), Select Crystal Structure Parameters, and Band Gap for Each Reported 3D Perovskite Structure^a^

Compound	α	Space group (RT)	Tilt	*D*	σ^2^	Avg. Bond Length (Å)	Unit Cell Vol./*Z* (Å^3^)	Band gap (eV)
CsPbI_3_^33^	0.851	*Pnam*	*a*^+^*b*^–^*b*^–^	0.0092	2.5422	3.1773	236.83	1.72
(MA)PbI_3_^34^	0.912	*I*4/*mcm*	*a*^0^*a*^0^*c*^+^	0.0006	0	3.1522	248.64	1.51
(FA)PbI_3_^35^	0.987	*Pm*3̅*m*	*a*^0^*a*^0^*a*^0^	0	0	3.181	256.9	1.41
(AZR)PbI_3_^36^	0.932	*Pm*3̅*m*	*a*^0^*a*^0^*a*^0^	0	0	3.1820	257.74	1.52
CsPbBr_3_^37^	0.862	*Pnma*	*a*^+^*b*^–^*b*^–^	0.0006	0.5659	2.9699	200.69	2.27
(MA)PbBr_3_^38^	0.927	*Pm*3̅*m*	*a*^0^*a*^0^*a*^0^	0	0	2.9664	208.82	2.23
(FA)PbBr_3_^39^	1.01	*Pm*3̅*m*	*a*^0^*a*^0^*a*^0^	0	0	3.0067	217.45	2.15
(AZR)PbBr_3_^36^	0.950	*Pm*3̅*m*	*a*^0^*a*^0^*a*^0^	0	0	2.9869	213.19	2.27
(MHz)PbBr_3_^29^	1.03	*P*2_1_	*a*^+^*b*^0^*c*^0^	0.0262	301.17	3.0424	209.18	2.49
CsPbCl_3_^37^	0.870	*Pbnm*	*a*^+^*b*^–^*b*^–^	0.0027	0.4451	2.8309	175.52	2.91
(MA)PbCl_3_^40^	0.938	*Pm*3̅*m*	*a*^0^*a*^0^*a*^0^	0	0	2.8433	182.80	2.88
(FA)PbCl_3_^41^	1.02	*Pm*3̅*m*	*a*^0^*a*^0^*a*^0^	0	0	2.8689	188.91	3.00
(AZR)PbCl_3_^36^	0.961	*Pm*3̅*m*	*a*^0^*a*^0^*a*^0^	0	0	2.8805	191.20	2.99
(MHz)PbCl_3_^42^	1.05	*P*2_1_	distorted	0.0242	314.38	2.9249	184.80	3.14
CsSnI_3_^43^	0.890	*Pnam*	*a*^+^*b*^–^*b*^–^	0.0028	21.998	3.1143	232.37	1.3
(MA)SnI_3_^44^	0.954	*Pm*3̅*m*	*a*^0^*a*^0^*a*^0^	0	0	3.1217	243.37	1.21
(FA)SnI_3_^45^	1.03	*Pm*3̅*m*	*a*^0^*a*^0^*a*^0^	0	0	n/a	251.19	1.41
CsSnBr_3_^46^	0.905	*Pm*3̅*m*	*a*^0^*a*^0^*a*^0^	0	0	2.9021	195.55	1.9
(MA)SnBr_3_^47^	0.973	*Pm*3̅*m*	*a*^0^*a*^0^*a*^0^	0	0	2.9537	206.16	2.3
(FA)SnBr_3_^45^	1.06	*Pm*3̅*m*	*a*^0^*a*^0^*a*^0^	0	0	2.9931	214.51	2.63^48^
(MP)SnBr_3_^49^	n/a	*Pc*	distorted	0.1079	8.6323	3.0462	215.79	2.45
(TMA)SnBr_3_^30^	n/a	*P*2_1_	distorted	0.1710	48.448	3.2144	259.42	2.76^31^
CsSnCl_3_^50^	0.916	*Pm*3̅*m*	*a*^0^*a*^0^*a*^0^	0	0	2.752	166.7	2.88^48^
(MA)SnCl_3_^50^	0.987	*P*1	distorted	0.0926	72.264	2.8286	186.04	3.5^48^
(GA)SnCl_3_^51^	1.06	*Pbca*	distorted	0.1612	99.193	3.0191	208.65	n/a
(TMA)SnCl_3_^31^	n/a	*Cmc*2_1_	distorted	0.2061	36.117	3.1373	242.34	3.59
CsGeI_3_^52^	1.03	*R*3*m*	distorted	0.0838	32.102	3.0044	213.96	1.6
(MA)GeI_3_^52^	1.06	*R*3*m*	distorted	0.1083	44.875	3.1093	235.73	1.9
(FA)GeI_3_^52^	1.14	*R*3*m*	distorted	0.1338	74.410	3.1551	242.72	2.3
CsGeBr_3_^53^	0.95	*R*3*m*	distorted	0.103	19.83	2.825	178.8	2.38
(MA)GeBr_3_^54^	1.09	*R*3*m*	distorted	0.1412	24.693	2.9112	195.24	2.91
(FA)GeBr_3_^54^	1.18	*R*3*m*	distorted	0.1668	77.212	2.9971	207.42	3.13
CsGeCl_3_^53^	0.985	*R*3*m*	distorted	0.1368	8.6546	2.7202	160.45	3.43

a AZR = aziridinium, MHz = methylhydrazinium, MP = methylphosphonium, TMA = trimethylammonium, GA = Guanidinium.

Most MA based perovskite compounds (MA)PbX_3_ (X = Cl, Br)^38,40,55^ and (MA)SnX_3_ (X = Br, I)^44,47^ have favorable tolerance factors (Table 1) and as a result form the *a*^0^*a*^0^*a*^0^*Pm*3̅*m* structures (Figure 3a) which are examples of ideal, high symmetry perovskite structures. MA cations form two other perovskite structures with Pb and Sn that tilt and deviate from ideal structures: (MA)PbI_3_ has been reported in the *Fmmm*,^38^*I*4/*m*,^56^*I*4/*mcm*,^34,57,58^ and *I*4*cm*^12^ space groups. Twinning in these crystals presents a challenge in assigning the space group via single crystal X-ray diffraction.^38^ Rotational SHG measurements which map the symmetry of the structure suggest bulk (MA)PbI_3_ likely adopts the *I*4/*mcm* space group.^57^ (MA)PbI_3_ has a slightly tilted structure described as *a*^0^*a*^0^*c*^–^ (Figure 3b). The slight tilting along a single axis correlates with an α value of 0.91 in the middle of the other halide perovskites. In contrast, (MA)SnI_3_ adopts the *Pm*3̅*m* space group (*a*^0^*a*^0^*a*^0^) since α is larger (0.95), pushing it closer to the stretch limit and thus having no tilting. The other nonideal MA based halide perovskite is (MA)SnCl_3_ (*P*1, Figure 3d),^50^ which can be described as a distorted perovskite with large σ^2^ and *D* values (Table 1). We can deduce that the lone pair of Sn^2+^ (5s^2^)^46^ in combination with the short Cl bonds cause the distortions in the (MA)SnCl_3_ structure, not predicted by the α and μ.^59^

The slightly larger formamidinium (FA) cation forms (FA)PbX_3_ (X = Cl, Br) and (FA)SnX_3_ (X = Br, I) perovskite structures in the *Pm*3̅*m* space group with no tilts (*a*^0^*a*^0^*a*^0^).^39,41,45,60^ These structures all form halide perovskites in spite of having a slightly unfavorable α > 1 (Table 1), which demonstrates the empirical nature of α. However, (FA)MI_3_ (M = Sn, Pb) that have α close to 1 are a metastable perovskite phase (*Pm*3̅*m, a*^0^*a*^0^*a*^0^) at room temperature.^35,61,62^ Under ambient conditions, (FA)PbI_3_ converts to a thermodynamically stable nonperovskite yellow phase, indicating the structural instability of these compounds.^61^ FA based perovskites are an excellent example of halide perovskites existing at the stretch limit, where the large FA cation causes MX_6_ octahedra in the structure to adopt the *Pm*3̅*m*, *a*^0^*a*^0^*a*^0^ structure with no tilting.

Cs^+^ is the primary alkali A-site cation found in the crystal structures of inorganic halide perovskites, although solid solutions containing Rb^+^ have been reported.^37,63^ At RT, CsPbX_3_ (X = Cl, Br) forms a tilted structure in the *Pbnm* (no. 62) space group.^37^ CsPbI_3_ crystallizes in the *Pm*3̅*m* space group above 583 K which can be quench cooled to a RT metastable perovskite phase in the *Pnam* space group (no. 62).^33^ The RT structures of CsPbX_3_ (X = Cl, Br, I) and CsSnI_3_ can be considered to be tilted perovskites with a Glazer notation of *a*^+^*b*^–^*b*^–^ (Figure 3c). This tilting is induced by the smaller Cs^+^ A-site cation leading to α < 0.9; thus the structures approach the tilt limit. CsMI_3_ (M = Sn, Pb) are unstable at RT and without stabilization will convert to a nonperovskite structure.^12,43^ CsSnX_3_ (X = Cl, Br) have α > 0.9 which is larger than their Pb counterparts and as a result fit into the *Pm*3̅*m* nontilted space group.^46,64^

Germanium is smaller than Sn and Pb and has more pronounced lone pair (4s^2^) effects. (A)GeI_3_ (A = MA, FA)^52^ and CsGeX_3_ (X = Cl, Br, I)^52,65^ crystallize in the polar *R*3*m* space group (Figure 3d). The GeX_6_^4–^ octahedra are trigonally distorted with three elongated Ge–X bonds. With increasing A-site cation size in the CsGeI_3_, MAGeI_3_, and FAGeI_3_ series, the Ge–I bonds elongate, resulting in a larger unit cell volume and higher *D* values (Table 1). The *R*3*m* symmetry of Ge-based perovskites is caused by the small Ge with prominent lone pair effects and does not vary with A-site cation.^66^ The same trends and symmetry occur true for (A)GeBr_3_ (A = Cs^+^, MA, FA) and (MA)GeCl_3_.^54,67,68^ All (A)GeX_3_ compounds have higher σ^2^ and *D* values than their Pb and Sn counterparts (Table 1).

Notably, exotic cations larger than FA have been incorporated into the 3D halide perovskite structures. Methylhydrazinium (MHz) has been incorporated into (MHz)PbCl_3_^42^ and (MHz)PbBr_3_^29^ which both crystallize in the noncentrosymmetric *P*2_1_ space group (Figure 3e).^69^ Upon heating, these compounds undergo a phase change to structures with higher symmetry *Pm*3̅*m* (Br, 418 K) and *Pb*2_1_*m* (Cl, 342 K) space groups. The MHz cation highlights the unique effect of the A-site cation on the symmetry reduction due to not only the large size of the cation but also a contribution from coordination bonds of the terminal nitrogen groups in these large cations. In the RT crystal structure of (MHz)PbCl_3_, the nitrogen coordinates selectively with specific lead sites leading to opposite distortions in every other layer and resulting in the low symmetry *P*2_1_ space group at RT. Moreover, methylphosphonium (MP) and trimethylammonium (TMA) have been reported in distorted, Sn-based perovskite structures. (MP)SnBr_3_ crystallizes in the *Pc* space group.^49^ (TMA)SnBr_3_ and (TMA)SnCl_3_ crystallize in the *P*2_1_ (Br) and *Cmc*2_1_ (Cl) space groups (Figure 3f).^31,70^ TMA has also been found in (TMA)GeCl_3_ adopting the *Pnma* or *Pna*2_1_ space group at RT, though no crystal structure is available to us.^71,72^ Aziridinium (AZR) has been reported to be incorporated into (AZR)PbX_3_ (X = Cl, Br, I) which crystallize in the *Pm*3̅*m* at RT.^36,73^ It should be noted that the AZR cation is a highly reactive species due to the strain in a three-atom ring and undergoes rapid nucleophilic ring openings among other chemistries. While not explicitly studied to our knowledge, these AZR cations and their perovskites are likely prone to degradation via, for example, hydrolysis of AZR to the hydroxylammonium cation in the presence of water.

These low symmetry compounds are best described by the large distortions that such oversized A-site cations induce in the perovskite structure. All of these 3D halide perovskites incorporating large A-site cations have unusually large *D* and σ^2^ due to the strain imposed on the M–X framework, which must contort to accommodate the oversized A-site cations. This distortion can be seen in Figure 3f, where the octahedron has extremely elongated bonds, which are at the edge of what is considered octahedral coordination. It is also worth noting the specific symmetry element of a 2_1_ screw axis introduced in (MHz)PbX_3_ (X = Cl, Br) and (TMA)SnBr_3_ has become of interest for its helical nature.^74^

Briefly, there have been select demonstrations of fluoride based halide perovskites, specifically CsPbF_3_^75^ demonstrated experimentally and others explored computationally.^76−78^ Other reports on related CsMF_3_ structures are nonperovskite phases.^79^ These compounds are an underexplored area of halide perovskite research and may not be promising as semiconductors, though creative research using alkaline metals as the metal site in the fluoride perovskite may lead to large ionic conductivities as have been observed previously.^80^

It should also be noted that we have focused on the room temperature phases of these 3D halide perovskites. Yet, there is extensive work on the temperature induced phase changes of halide perovskites. Usually the crystal symmetry is reduced as a halide perovskite is cooled (either from elevated temperatures or room temperature), which undergoes phase changes from the highest symmetry *Pm*3̅*m* to lower symmetry phases as outlined by a recent review.^81^

### Exotic and Perovskite-like Structures

In addition to the typical AMX_3_ perovskite structures, there has been an interest in identifying structures related to this motif for enhanced functionality or tailoring of the properties. One particular class is the A_2_MM′X_6_ (M = monovalent metal, M′ = trivalent metal) double perovskites (sometimes referred to as elpasolite based on the mineral K_2_NaAlF_6_).^82^ These double perovskites can be broken into two classes: single metal multivalent and mixed-metal multivalent double perovskites. Some examples of single metal systems include Cs_2_Tl^1+^Tl^3+^Cl_6_^83^ and Cs_2_Au^1+^Au^3+^Cl_6_.^84^ A contemporary example of a mixed metal double perovskite is Cs_2_AgBiBr_6_ that grows in the *Fm*3̅*m* space group^85^ and other examples recently reported.^86−88^ These double perovskites have ordering due to the ionic size difference between Ag and Bi sites (sometimes deemed as “ordered double perovskites”). In these double perovskites, alkali metals are routinely utilized as an A-site cation. However, recent work has gone into making hybrid organic inorganic double perovskites using MA cations.^89^ A more comprehensive discussion of double perovskites can be found here.^82^

Other unique perovskite-like structure types include the so-called “hollow” perovskites which incorporate a large divalent ethylenediammonium (en) cation.^90^ In these structures, the divalent en cation not only occupies the A-site cation position but also induces metal and halide vacancies forming structures with the formula (A)_1–*x*_(*en*)_*x*_(M)_1–0.7*x*_(X)_3–0.4*x*_.^91−93^ Because the M–X–M connectivity is not always completely corner-sharing in these structures, they are considered “perovskitoids” or perovskite-like; therefore, it could be debated if the associated organic cations should be called “A-site cations” in the strictest sense. One can consider these examples as at the boundary of the A-site cation chemistry in metal halide perovskites. Another interesting example is the incorporation of zwitterionic cystamine, with a formal negative charge on the sulfide and a positive charge on the amine. This forms a unique (NH_3_(CH_2_)_2_S)PbX_2_ (X = Cl, Br) perovskite structure, where the sulfur anion occupies the site of one halide as determined by X-ray pair distribution function analysis.^93^

### A-Site Cation Orientation and Motion

FA and MA cations are highly disordered in the A-site cavity of AMX_3_ at RT. Solid-state NMR can effectively capture this dynamic cation position and show that the MA cation in (MA)PbX_3_ has no fixed position within the A-site cavity.^94,95^ Instead, the NH_3_^+^ group reorients inside the cavity, interacting with the different halides in the lattice through hydrogen bonding, while the CH_3_ group does not interact with the lattice. These are isotropic reorientations with activation energies on the order of 6–12 kJ/mol.^96^ The activation energy of the reorientation decreases in the order of halide (Cl > Br > I), which can be attributed to the strength of the NH_3_···X hydrogen bonding. Based on 2D vibrational spectroscopy combined with ab initio molecular dynamics simulations, there are likely two reorientations occurring: a fast, local wobbling-in-a-cone motion (Figure 3g) and a slower 90° jump (Figure 3h).^32,95^ The fast wobbling occurs on the 0.2–0.4 ps time scale, while the jump reorientation is 3 ps. Other reports based on neutron scattering suggest time scales are up to 14 ps.^97^ The discrepancy is described in list of reorientation times presented by Gallop et al.^32^ The FA cation behaves similarly, exhibiting rapid wobbling within a cone along with reorientation jumps.^98^ The rotation about the N–N axis of FA has an energy barrier of 21 meV and occurs on the 8 ps time scale.^99^ For further discussion on the cation dynamics in halide perovskites, we refer readers to recent reviews.^63,100^ The cation reorientation at rapid time scales is important for the (high symmetry) space groups assigned above as the disordered MA and FA cations modeled in the crystallographic structures are likely the most accurate assignments reflective of the average (i.e., rotating) A-site cation. Another interesting observation shown through neutron inelastic spectroscopy and modeling is that the cations can have local ordering akin to ferroelectric domains that is coupled to dynamic octahedral tilts, suggesting the molecular rotations are coupled to the inorganic lattice instead of completely free rotations.^101^

### A-Site Cation Alloying

**Figure 4:**
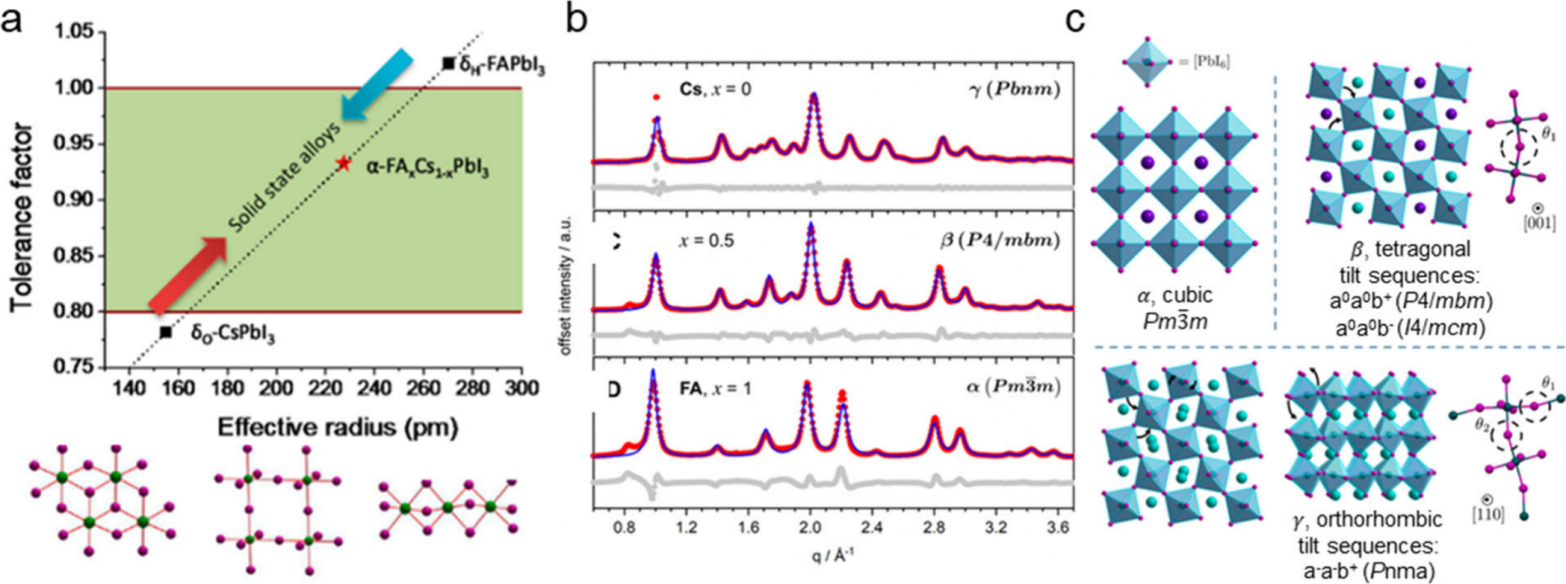
Stabilization of metastable perovskite phases via A-site cation alloying and large A-site cation 2D RP perovskites. (a) Tolerance factor versus effective A-site cation radius for solid-solutions of (Cs_1–*x*_FA_*x*_)PbI_3_ with the corresponding crystal structures of the perovskite and nonperovskite phases below. (b) Rietveld refinement of pure CsPbI_3_, mixed (Cs_0.5_FA_0.5_)PbI_3_, and (FA)PbI_3_ nanocrystals with diffraction patterns. (c) Structures corresponding to panel b. Panel a is reproduced or adapted with permission from (102). Copyright 2016 American Chemical Society. Panels b and c are reproduced or adapted with permission from (103). Copyright 2020 American Chemical Society.

Select 3D halide perovskite phases such as (A)PbI_3_ (A= Cs^+^, FA) are unstable as the perovskite phase at RT. However, these perovskites are very attractive for PV applications and can be stabilized via A-site cation alloying, among other techniques. An early example of alloying for stabilization was shown in (Cs_1–*x*_FA_*x*_)PbI_3_ thin films.^102^ By alloying Cs–FA, the tolerance factor was effectively tuned between the small Cs and large FA structures (Figure 4a) and resulted in stable halide perovskite materials. Grazing incidence wide-angle X-ray scattering on nanocrystal films further revealed that tuning the FA/Cs ratio in (Cs_1–*x*_FA_*x*_)PbI_3_ changed the tilting and symmetry of the crystal structure (Figure 4b).^103,104^ At low Cs content (*x* > 0.7), the high symmetry *Pm*3̅*m* structure is favored (Figure 4c). By incorporation of more FA (0.7 > *x* > 0.2), a lower symmetry *P*4/*mbm* structure is formed, analogous to what is observed in (MA)PbI_3_ (*I*4/*mcm*). At high Cs concentrations (*x* > 0.2), the structure adopts the tilted *Pbnm* space group. Other examples of A-site cation alloying at room temperature do not show changes in symmetry or tilting, but simple expansion/contraction of the unit cell based on the cation size.^37,105−108^ It should also be noted that there is evidence of A-site cation mobility in these alloyed structures under external stimuli, such as an electrical bias. While A-site cations are not as mobile as halide anions,^104,109^ over the time scale of device operation and under the external stimuli of light soaking and bias the A-site cations do begin to phase segregate.^110^ A-site cation alloying and more generally compositional engineering including the metal and halide ions has proven to be a key component of making high efficiency perovskite solar cells, including tandem perovskite solar cells, and is described in detail in many recent reviews focused on perovskite solar cells.^19,111−113^

### A-Site Cations in 2D RP Perovskites

**Figure 5:**
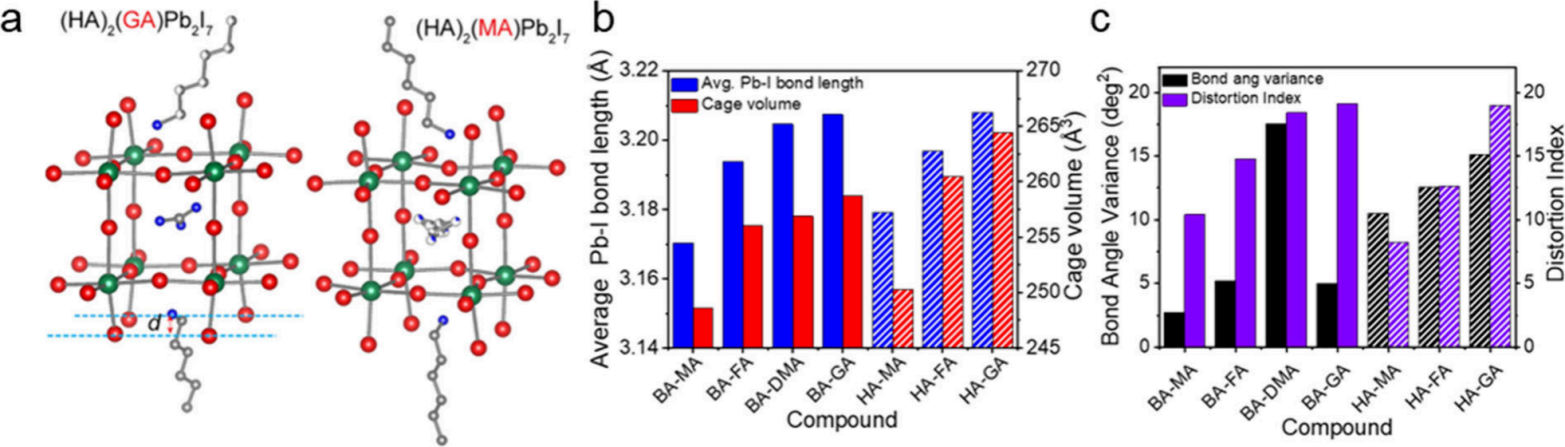
(a) Crystal structure of (HA)_2_(GA)PbI_7_ compared to that of (HA)_2_(MA)PbI_7_. (b) Average Pb–I bond length and cage volume of select large A-site cation RP perovskites showing an increase in length/size of inorganic framework with cation size. (c) Bond angle variance and distortion of RP perovskites showing the increase in distortion index with A-site cation size. BA = butylammonium, HA = hexylammonium, DMA = dimethylammonium, and GA = guandidinium. Panel a is reproduced or adapted with permission from (114). Copyright 2019 American Chemical Society. Panels b and c are reproduced or adapted with permission from (124). Copyright 2020 American Chemical Society.

2D (A′)_2_(A)_*n*−1_M_*n*_X_3*n*+1_ Ruddlesden–Popper (RP) perovskites can accommodate a larger range of A-site cations than AMX_3_ halide perovskites.^114−117^ In RP perovskites, the layers of the inorganic M–X framework are interceded by flexible long chain ammonium spacer cations (A′),^17^ which stabilizes the perovskite cages of *n* > 1 RP perovskites that incorporate large A-site cations into the 12-coordinate A-site pocket. The large guanidinium (GA) cation has been incorporated into (A′)(GA)Pb_2_I_7_ with A′ = *n*-butylammonium (BA),^116^*n*-pentylammonium (PA),^118,119^ and *n*-hexylammonium (HA)^114^ and confirmed by single-crystal X-ray crystallography. The GA cation alone (*i.e*., if attempted to grow as an AMX_3_ perovskite) forms a nonperovskite structure lacking the 12-coordinate A-site cavity.^114^ In these RP structures with a large A-site cation, the volume of the A-site cavity is dramatically increased relative to the corresponding (A′)(MA)Pb_2_I_7_ (Figure 5a). In (HA)_2_(A)Pb_2_I_7_ with A = MA and GA structure, there is a clear increase in Pb–I bond length and A-site cavity volume when the larger GA cation is incorporated, which matches the trend for other A′ cations such as PA.^118^ Furthermore, the large GA cation distorts the PbI_6_^4–^ octahedra, resulting in larger σ^2^ and *D* relative to (A′)(MA)Pb_2_I_7_ structures (Figure 5b,c). Similarly, other *n* = 2 RP perovskite compounds with larger cations, such as (A′)(FA)Pb_2_I_7_ (A′ = BA,^116^ HA^120^) and (A′)_2_(DMA)Pb_2_I_7_ (A′ = BA,^116^ PA^121^), exhibit elongated Pb–I bonds and larger distortion relative to RP perovskites with MA cations, though to a lesser extent than GA (Figure 5b,c). Comprehensive structural analysis comparing across a series of six A-cations (MA, FA, DMA, EA, GA, and AA) in (PA)_2_(A)Pb_2_I_7_ demonstrated the significance of the shape, polarity, and hydrogen bonding interactions of the A-cations, beyond simply cation size, in dictating the local distortions and expansion of the perovskite cage which has consequences on the structural symmetry.^122^ Similar to the 3D perovskites, enhanced *ns*^2^ lone pair expression in germanium and tin (A′)_2_(A)X_2_I_7_ RP perovskites can lead to more significant octahedral distortions across the A-cation^66^ that are the most pronounced for germanium analogues,^123^ but are further dependent on the choice of A′.

Higher *n*-value RP perovskite structures with large A-site cations follow the same trend: the Pb–I bonds in (A′)_2_(EA)_2_Pb_3_I_10_ (EA = ethylammonium, A′ = BA,^124,125^ PA,^126^ HA^127^) are elongated and induce a larger A-site cavity compared to (BA)_2_(MA)_2_Pb_3_I_10_. Moreover, the changes in Pb–I bonding in these *n* = 3 RP perovskite structures depend on whether the octahedra is in the inner layer or the two outer layers of PbI_6_^4–^ octahedra. The inner layer has significantly more distorted octahedra, as it is templated by two EA cations on both sides with large steric interactions. Whereas the outer layers are templated by one EA cation and a more flexible BA cation, leading to less distorted octahedra. Similar to the iodides, the smaller bromide based RP structures also feature increasing *D*, σ^2^, bond lengths, and cage volume as the A-site cation size increases. (BA)_2_(A)Pb_2_Br_7_ (A= Cs,^128,129^ MA,^130^ FA^131^) form in the *Cmc*2_1_ space group at RT. Moving to the larger EA cation, multiple (A′)(EA)_*n–*1_Pb_*n*_Br_3*n*+1_ structures have been demonstrated with the A′ spacer cation varying from EA,^132^ BA,^133^ isobutylammonium (IBA),^134^ and 4-aminomethyl-1-cyclohexanecarboxylate.^135^ The structure (EA)_4_Pb_3_X_10_ (X = Cl, Br) form a unique RP layered structure where the EA cation serves as both the A-site cation as well as the spacer cation.^132,136^ A unique, a cyclic A-site cation 1,2,4-triazolium (Tz) has been incorporated into (IPA)_2_(Tz)_*n*−1_Pb_*n*_Br_3*n*+1_ (*n* = 2, 3) with large distortions in the octahedra.^137^ Other large cations such as DMA and MHz have also been introduced to the bromide RP halide perovskites.^138^ In addition, 2D Dion–Jacobson phase with divalent spacer cations (A′) interceding the layers has also had large A-site cations incorporated.^139−141^ In general, incorporation of large A-site cations into the RP halide perovskites promotes structural changes similar to those of the AMX_3_ halide perovskites with larger A-site cation, where the octahedra stretch and distort to accommodate the size of the cation. The ability to incorporate even larger cations in the 2D RP halides compared to 3D perovskites is a result of the compressive strain accommodated by the spacer cation being flexible.^114,116^

### Influence of A-Site Cation on Optical Properties

A recent review on the growth of single crystal halide perovskites shows the bandgap energy (E_g_) slightly varies with the growth conditions of the compound, yet general trends can be discussed as they relate the crystal structure to optical properties.^142^ The series Cl > Br > I and Ge > Pb > Sn and are ordered from largest to smallest bandgap energy (E_g_).^12,52,143^ The A-site cation does not significantly contribute to the density of states making up the conduction and valence band but indirectly affects the band gap and optoelectronic properties by templating the bonding of the M–X framework. Tilted structures such as CsPbI_3_ have a larger E_g_ (1.72 eV) relative to the nontilted structure (FA)PbI_3_ (1.41 eV).^144,145^ This effect is the result of a shift in the overlap of the M and X orbitals due to the tilting.^146,147^ Based on DFT calculations, changes in symmetry do not significantly alter the band dispersion, only the band gap.^148^

**Figure 6:**
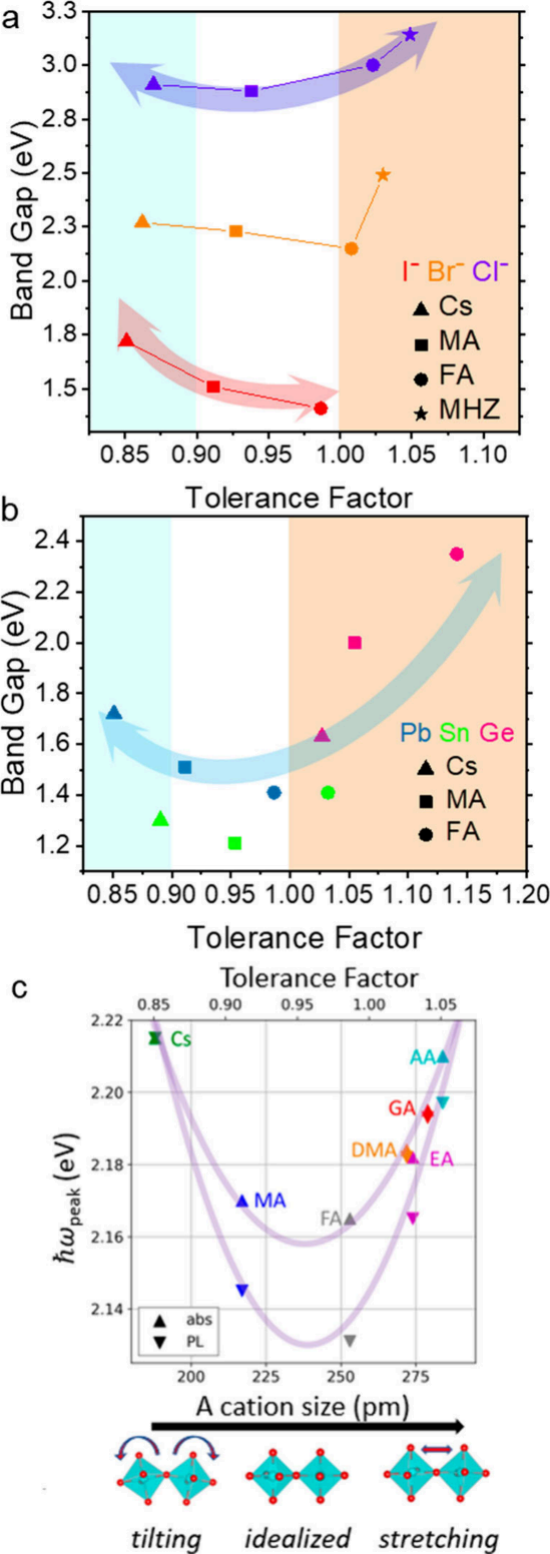
Relationship of band gap to Goldsmidt tolerance factor in various 3D AMX_3_ perovskites. (a) Band gap versus tolerance factor of APbX_3_ showing the band gap decrease with larger tolerance factor for each halide series until reaching a stretching limit (∼α > 1), where the band gap increases. (b) Band gap vs tolerance factor for AMI_3_ with a similar downward sloping trend for ∼ α < 0.9, followed by increasing band gap at ∼α > 1 (shaded for clarity). (c) Relationship of A-site cation size and absorbance/photoluminescence in nanocrystals of large A-site cation *n* = 2 RP perovskites. Panel c is reproduced or adapted with permission from (115). Copyright 2020 American Chemical Society.

The E_g_ of alloyed (Cs_1–*x*_Rb_*x*_)PbX_3_ (X = Cl, Br) and (FA_1–*x*_MA_*x*_)PbI_3_ decrease linearly as the tolerance factor increases (i.e., the average A-site cation size gets larger).^37,149^ Focusing the trendlines on a single metal and halide (constant *r*_*M*_ and *r*_*X*_) and comparing the E_g_ of APbX_3_ (X = Cl, Br, I) versus the tolerance factor, we can observe a bowing trend with increasing A-site cation size (Figure 6a). In the APbCl_3_ series, the tilted CsPbCl_3_ has a wider gap than the less tilted (MA)PbCl_3_ which displays the narrowest E_g_. The larger stretch limit (FA)PbCl_3_ structure has a higher E_g_ due to the decreased M–X overlap as a result of the elongated Pb–Cl bonds. The even larger (MHz)PbCl_3_ has the largest E_g_, due to even more elongated bonds and distortion in the octahedra. This trend seems consistent with the APbBr_3_ and APbI_3_ series, though there are fewer data points to compare. Plotting the band gap versus tolerance factor of AMI_3_ (constant *r*_*I*_) for different M (Pb, Sn, Ge) and A-site cations reveals a similar trend (Figure 6b). The perovskite structures with a moderate tolerance factor (0.9–1.0, the wide band in the middle) have the smallest E_g_, and the E_g_ increases below and above this range. For α < 0.9 (small A-site cation for the structure), tilting will raise the E_g_. At α > 1 (A-site cation too big for the structure), the M–X bonds are elongated causing an increase in E_g_. In moderate range (0.9 < α < 1.0) the curve is flat. This trend is consistent with the observations in RP perovskites which have an expanded range of A-site cation sizes. Figure 6c shows nanocrystals of RP perovskites (HA)_2_(A)Pb_2_I_7_ (HA = hexylammonium) with various A-site cations ranging from small Cs to large guanidinium (GA) and acetamidinium (AA) cations. There is a parabolic trend between the bandgap and cation size similar to the trends with 3D perovskite and tolerance factor, but showing more definitively the effect of the Pb–I stretching (induced by large A-cations) increasing the band gap. An interesting observation is that by external pressure applied, which effectively shortens Pb–I bond distance, the band gap begins to decrease.^150,151^ This shows that elongation of the bond distances increases the band gap, while compression decreases the band gap, effectively getting both ends of the spectrum. A more comprehensive review of halide perovskites under pressure can be found here.^152^

## Conclusions

Metal halide perovskites are in the process of being commercialized as solar absorbers and are promising materials for other optoelectronic applications owing in part to their structural tunability. As a comparatively less studied and discussed compositional tuning approach, the A-site cation provides an alternative way to tune the perovskite crystal structures and bonding and thus influences the physical properties of the materials. In this review, we have provided a comprehensive summary of 3D AMX_3_ halide perovskite structures with a diverse set of A-site cations with a focus on empirical results demonstrating the influence of the A-site cation on the structural distortion and overall perovskite structure. Further discussion on A-site cation motion, alloying, and expanded A-site cation compositions in 2D RP perovskites is provided to further explain the impact of the A-site cations. The crystal structure is then correlated with band gap to understand the relationship of the halide perovskite structure on optical properties. Using A-site cations together with other compositional changes to access refined structure and property tuning could drive continued progress in the chemistry, properties, and applications of halide perovskites and broader metal halide materials.

In addition to enhancing photophysical properties for improved optoelectronic devices already demonstrated by current literature, many areas of research can be aided by further tuning the A-site cations in halide perovskites.^153^ This includes enhancing nonlinear (second harmonic generation, ferroelectricity, etc.) properties and Rashba band splitting,^59,122,123^ which are dependent on the symmetry of the material, in particular materials with noncentrosymmetric polar space groups.^154,155^ In addition, many phenomena exhibited by halide perovskite materials could be potentially useful for computational information science, including superfluorescence,^156^ control over spin polarization,^7^ and single photon emitters.^157^ While not explicitly dependent on structural symmetry, these properties can be enhanced or modulated by utilizing new compositions enabled by A-site cation chemistry. Third, it appears there has been a significant amount of research into the narrow band gap lead and tin iodide based perovskites owing to their utility in solar cells. However, since many of these potential applications beyond solar cells do not require a narrow band gap (as is the case for solar), it is worthwhile for researchers to further explore the chloride and bromide based perovskites, which often exhibit more diverse and unique structures enabled by large A-site cations (such as the interesting *P*2_1_ (TMA)SnBr_3_ structures^70^). At the core of these potential applications, exploring new halide perovskite structures enabled by A-site cations, developing new 2D RP perovskite structures with expanded A-site cations, and the resulting enhanced structural control in halide perovskites will aid in future endeavors of halide perovskite materials research for broad applications.
